# Cytomorphological Spectrum and Diagnostic Utility of Tzanck Smear Cytology in Vesiculobullous Skin Lesions: A Cross-Sectional Study From a Tertiary Care Center in Eastern Uttar Pradesh, India

**DOI:** 10.7759/cureus.96803

**Published:** 2025-11-13

**Authors:** Priyanka Gautam, Brijnandan Gupta, Divya Singh, Sunil K Gupta, Shiwangi Rana, Mimna V M

**Affiliations:** 1 Department of Pathology and Laboratory Medicine, All India Institute of Medical Sciences, Gorakhpur, Gorakhpur, IND; 2 Department of Pathology, Sanjay Gandhi Postgraduate Institute of Medical Sciences, Lucknow, IND; 3 Department of Dermatology, All India Institute of Medical Sciences, Gorakhpur, Gorakhpur, IND

**Keywords:** cytology, dermatopathology, diagnostic dermatology, herpetic infection, pemphigus vulgaris, tzanck smear, vesiculobullous lesions

## Abstract

Background

Tzanck smear cytology is a simple, rapid, cost-effective, and minimally invasive diagnostic tool used in evaluating vesiculobullous skin lesions. It is beneficial in the early diagnosis of herpetic infections and autoimmune blistering diseases such as pemphigus vulgaris, where acantholytic (Tzanck) cells may be observed. However, interpretation must be made in conjunction with clinical history and examination findings.

Aim

This study aims to assess the diagnostic utility and limitations of Tzanck smear cytology in the evaluation of vesiculobullous skin disorders in correlation with clinical findings, with a focus on its relevance in the Indian population. A limited amount of research has been conducted in Indian literature regarding the utility and limitations of Tzanck smear cytology in treating different types of dermatological disorders.

Methods

A cross-sectional study was conducted at the Dermatology outpatient department and the Pathology department of a newly established tertiary care center in Eastern Uttar Pradesh, India. Over a six-month period (June 2023-November 2023), all patients presenting with at least one vesiculobullous skin lesion underwent Tzanck smear examination. Smears were prepared using standard techniques, stained with May-Grünwald Giemsa (MGG), and evaluated microscopically. Cytological findings were correlated with patient history and clinical diagnosis.

Results

A total of 30 patients were included in the study. Tzanck smears revealed diagnostic findings in 10 cases, with pemphigus vulgaris being the most common (n = 8), followed by herpetic infections (n = 2). The remaining 20 cases showed non-specific or negative findings and required further evaluation through histopathological biopsy.

Conclusion

Tzanck smear cytology can expedite clinical decision-making, reduce the need for invasive procedures, and be effectively used in conjunction with histopathological evaluation, particularly in resource-limited settings. Tzanck smear can be an effective tool as a preliminary step in evaluating vesiculobullous lesions of skin in busy tertiary care centers to filter out benign diagnoses, specifically bullous pemphigoid, as well as herpes vesicles.

## Introduction

The Tzanck smear is a cytological technique first introduced in 1947 by French physician Arnault Tzanck as a diagnostic tool for vesiculobullous conditions, particularly herpes simplex virus (HSV) infections [1,2]. Since its inception, the Tzanck smear has been employed in the evaluation of a broad spectrum of dermatological disorders, including vesiculobullous, erosive, tumoral, and granulomatous lesions [3-5]. Its diagnostic value in herpes simplex and herpes zoster infections was further validated by Blank et al. in 1951 [6].

The technique is valued for being rapid, simple, minimally invasive, and cost-effective, making it especially useful in settings with limited resources. Despite its simplicity, accurate interpretation requires clinical expertise, as cytological findings must be correlated with the patient's history and clinical presentation [7]. The procedure involves scraping the base of a fresh vesicle or erosion, staining the collected cells (commonly with May-Grünwald Giemsa (MGG)), and examining them under light microscopy.

In the context of herpetic infections, the identification of multinucleated giant cells on a Tzanck smear can facilitate early diagnosis, even when viral cultures are inconclusive. Similarly, in autoimmune blistering disorders such as pemphigus vulgaris (PV), the presence of acantholytic (Tzanck) cells provides supportive evidence for diagnosis.

Despite its wide applicability, the use of Tzanck smear cytology remains underreported in Indian dermatological literature, and its diagnostic potential may be underestimated in clinical practice. There is a need to better understand its utility, particularly in tertiary care settings, where a high patient load necessitates fast and reliable preliminary diagnostic tools. The present study aims to evaluate the diagnostic utility of Tzanck smear cytology in patients with vesiculobullous skin lesions and to correlate cytological findings with clinical data, thereby assessing its relevance and limitations in routine dermatological practice.

## Materials and methods

Study design

This was a cross-sectional observational study conducted at a newly established tertiary care center, All India Institute of Medical Sciences, Gorakhpur, India, from June 2023 to November 2023.

Inclusion criteria

The inclusion criteria include patients of all age groups and genders, presenting to the dermatology outpatient department with at least one vesiculobullous lesion, and consenting to undergo a Tzanck smear.

Exclusion criteria

Patients with secondary infections obscuring morphology, previously treated cases on systemic therapy, or those unwilling to provide consent were excluded from the study.

Procedure

Smears were obtained from the base of vesicles or bullae using conventional methods. In the case of vesicles and bullae, a scalpel was used to cut along one side and then fold back. Swabs were then carefully taken from the fluid contents. Next, a scalpel was used to scrape the floor of the lesion. An erosion was detected by scraping the advancing border. After obtaining the cellular material, a thin layer was spread over a clean glass slide. Slides were air-dried and stained using MGG stain. Microscopic examination was performed under light microscopy by trained pathologists to identify acantholytic (Tzanck) cells, multinucleated giant cells, inflammatory cells, and viral cytopathic changes. All smears were evaluated independently by two pathologists, and the final interpretation was made by consensus, thereby minimizing interobserver variability. Findings were recorded and correlated with clinical history and physical examination.

Data analysis

Data were tabulated using Microsoft Excel (Microsoft Corp., Redmond, WA, US). Diagnostic yield was calculated as the proportion of cases with cytological findings supporting a clinical diagnosis. Cases lacking definitive cytological diagnosis were referred for histopathological confirmation via biopsy.

## Results

**Figure 1:**
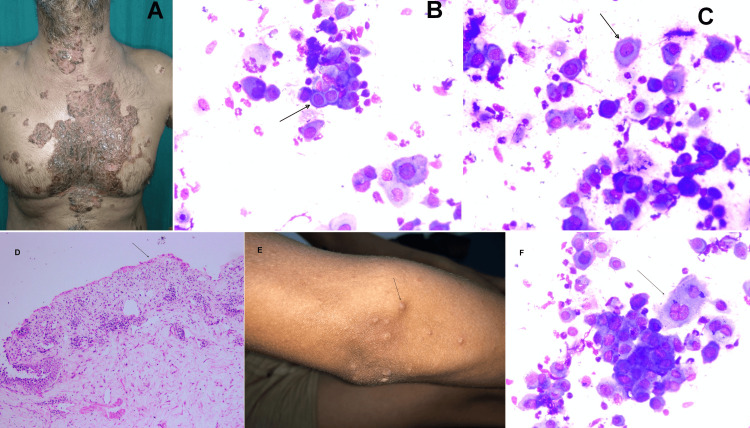
Clinical, cytology, and histology images of pemphigus vulgaris and varicella zoster (A) Clinical image of widespread lesions over multiple sites of the body suspected in the case of pemphigus vulgaris. (B, C) Acantholytic cells highlighted by an arrow in the Tzanck smear (MGG stain x400). (D) Biopsy image of pemphigus vulgaris showing classical histopathology of “row of tombstone” appearance (H&E stain x200). (E) Clinical image of a young male patient presenting with multiple tiny vesicles over the elbow region in a suspected case of varicella zoster. (F) Tzanck smear showing characteristic multinucleated giant cells with nuclear molding highlighted by an arrow (MGG stain x400). MGG: May-Grünwald Giemsa

A total of 30 cases were included in this study. The age of patients ranged from two to 76 years, with a mean age of 39.5 years. There was a female predominance with 19 females (63.3%) and 11 males (36.7%), yielding a male-to-female ratio of 0.57:1. The distribution of cases according to Tzanck smear diagnosis is shown in Table 1. Most patients presented with vesiculobullous lesions, and three patients diagnosed with PV showed mucosal involvement. In 12 cases, lesions were widespread, involving multiple areas of the body (Figure 1A).

**Table 1:** Details of Tzanck smear diagnosis

Sr. No.	Diagnosis	No. of cases
1	Immunobullous lesions; pemphigus vulgaris	6
2	Viral lesions	2
3	Acute inflammatory lesions	9
4	Non-specific	11
5	Inadequate	2

Out of the 30 Tzanck smears analyzed, eight cases showed cytological features suggestive of immunobullous disorders, specifically PV, while two cases were indicative of viral infections belonging to the herpes group. In eight of 30 cases (26.7%), Tzanck smear findings correlated with the clinical diagnosis. The detailed cytomorphological findings of different diagnostic categories are summarized in Table 2.

**Table 2:** Cytomorphological findings +: present; -: absent

Diagnosis	Subtype	Findings on cytomorphology					
		Acantholytic cells	Squamous cells	Neutrophils	Lymphocytes	Multinucleated giant cells	Histiocytes
Immunobullous lesions	Pemphigus vulgaris	+	+	+	+	+	-
Viral lesions	Varicella zoster	+	-	-	-	+	-
Acute inflammatory lesions		-	+	+	+	-	+
Non-specific features		-	+	+	+	-	-
Others		-	+	+	+	+	-

In PV, smears revealed typical acantholytic (Tzanck) cells, characterized by large, round to oval keratinocytes with hyperchromatic nuclei, perinuclear halo, and deeply basophilic cytoplasm (Figures 1B, 1C). One suspected case of varicella zoster (Figure 1E) demonstrated multinucleated giant cells (Figure 1F) of varying sizes (2-5 nuclei), with prominent nucleoli, deep basophilic cytoplasm, and scattered acantholytic cells. A suspected case of bullous pemphigoid showed occasional clusters of multinucleated giant cells along with keratinocytes and inflammatory cells.

Other suspected clinical diagnoses included pustular psoriasis, bullous pemphigoid, non-healing ulcers, herpes simplex, atopic dermatitis, and lichen planus, which did not show distinct cytological features (Table 3). Among the remaining cases, nine smears showed acute inflammatory infiltrate, predominantly numerous neutrophils with few lymphocytes and histiocytes, while 11 smears revealed keratinocytes, few inflammatory cells, anucleate squames, and bacterial colonies. Two smears were inadequate due to poor technical quality, making cell morphology unrecognizable.

**Table 3:** Comparison of clinical, cytopathological, histopathological, and serological findings HSV: herpes simplex virus

Patient No.	Age (years)	Sex	Clinical diagnosis	Cytopathological findings	Histopathological and serological findings
1	7	M	Chronic bullous disease of childhood (CBDC), insect bite hypersensitivity	Inadequate	Biopsy not done, patient responded to symptomatic treatment
2	60	M	Oral pemphigus	Acantholytic cells present, suggestive of pemphigus vulgaris	Dsg 1 & 3 +ve IgG, IgM suggestive of pemphigus vulgaris
3	43	M	Genital ulcer	Squamous cells+, bacterial colonies +	Herpes serology + IgG, IgM
4	35	F	Pemphigus vulgaris	Acantholytic cells present, suggestive of pemphigus vulgaris	Biopsy suggestive of pemphigus vulgaris
5	64	M	Pemphigus vulgaris	Acantholytic cells present, suggestive of pemphigus vulgaris	Biopsy not done
6	8	F	Pemphigus vulgaris	Squamous cells +	Lost to follow-up
7	25	F	Pemphigus vulgaris	Dense inflammation	Lost to follow-up
8	30	F	Pemphigus vulgaris	Predominantly neutrophils, with a few scattered anucleate squames	Lost to follow-up
9	15	F	Bullous lichen planus	Squamous cells, inflammatory cells	Biopsy non-specific, suggestive of chronic dermatitis
10	62	M	Bullous pemphigoid	Keratinocytes & inflammatory cells, multinucleated giant cells	Lost to follow-up
11	74	M	Bullous pemphigoid	Few scattered degenerated inflammatory cells	Biopsy inconclusive
12	16	M	Non-healing ulcer (glans penis)	Neutrophils and squamous cells	HSV serology negative
13	28	F	Pemphigus vulgaris	Acantholytic cells present, suggestive of pemphigus vulgaris	Dsg 1 & 3 +ve IgG, IgM suggestive of pemphigus vulgaris
14	20	M	Lichen planus	Few squamous cells	Biopsy suggestive of lichen planus
15	26	F	Varicella	Numerous acantholytic cells, multinucleated giant cells	Biopsy not done, responded to anti-viral treatment
16	72	F	Bullous pemphigoid	Numerous neutrophils and a few squamous cells	Biopsy showed a subepidermal split suggestive of bullous pemphigoid
17	63	F	Pemphigus vulgaris	Predominantly neutrophils and squamous cells	Lost to follow-up
18	24	F	Pustular psoriasis	Scattered neutrophils and keratinocytes	Biopsy suggestive of pustular psoriasis
19	37	F	Miliaria rubra	Predominantly neutrophils and mature squamous cells	Final diagnosis miliaria rubra
20	46	F	Pemphigus foliaceus	Acantholytic cells present, occasional multinucleated giant cells	Biopsy suggestive of pemphigus
21	56	F	Chicken pox, herpes	Keratinocytes, neutrophils, few histiocytes, and lymphocytes	Patient responded to acyclovir
22	33	M	Pemphigus vulgaris	Keratinocytes, neutrophils, few histiocytes and lymphocytes	Lost to follow-up
23	18	F	Dermatitis herpetiformis	Neutrophils, lymphocytes, histiocytes, and occasional keratinocytes	Biopsy inconclusive, TTG positive
24	69	F	Pemphigus vulgaris	Acantholytic cells present, multinucleated giant cells	Skin biopsy inconclusive herpes serology + IgG, IgM
25	58	F	Pemphigus foliaceus	Squamous cells	Lost to follow-up
26	31	F	Pemphigus foliaceus	Few acantholytic cells present	Biopsy suggestive of pemphigus
27	66	F	Bullous pemphigoid	Few scattered squamous cells and neutrophils	Biopsy not done
28	3	M	Insect bite hypersensitivity	Dense inflammation, squamous cells	Biopsy not done
29	2	F	CBDC	Squamous cells	Biopsy subepidermal split seen, suggestive of CBDC
30	18	M	Psoriasis	Squamous cells	Lost to follow-up

The correlation of clinical, cytopathological, histopathological, and serological findings is presented in Table 3. Histopathological examination was performed in 11 out of 30 cases suspected of having immunobullous lesions. Of these, four cases showed histopathological findings consistent with PV (Figure 1D), correlating with the clinical diagnosis. Two cases confirmed the clinical diagnoses of lichen planus and pustular psoriasis, respectively. Three biopsies were inconclusive, while one biopsy was non-specific and suggestive of chronic dermatitis. In addition, one suspected case of chronic bullous disease of childhood (CBDC) was confirmed on biopsy.

Serological evaluation revealed that two patients tested positive for desmoglein 1 (DSG1) and desmoglein 3 (DSG3) autoantibodies, supporting the diagnosis of PV. Two cases showed positive herpes serology, whereas one case tested negative. A total of nine patients (30%) were lost to follow-up, which limited further diagnostic confirmation.

## Discussion

The utility of the Tzanck smear has expanded beyond its traditional role in diagnosing pemphigus and herpes infections. It is now recognized as a valuable tool in diagnosing a variety of cutaneous infections, including molluscum contagiosum, candidiasis, and leishmaniasis, often obviating the need for biopsy confirmation [8]. Durdu et al. further demonstrated its value across erosive, vesicular, bullous, and pustular lesions, underscoring the breadth of its diagnostic potential [2]. However, the diagnostic accuracy of this well-established yet simple technique relies heavily on interpreting smear findings within the appropriate clinical context.

The fundamental basis of the Tzanck smear is the identification of acantholysis-a pathological process characterized by the loss of intercellular connections between keratinocytes. The term acantholysis derives from the Greek words akantha (thorn or prickle) and lysis (loosening) [9,10]. During acantholysis, the breakdown of desmosomal junctions results in the formation of clefts, vesicles, or bullae within the epidermis. The affected keratinocytes lose cohesion and adopt a rounded morphology. The hallmark cytological feature, known as the Tzanck cell, is a large, rounded keratinocyte exhibiting a hyperchromatic nucleus with a perinuclear halo and deeply basophilic cytoplasm [5]. Also described are variations in acantholytic morphology, such as amoeboid projections and dyskeratotic changes, highlighting the potential pitfalls of cytological interpretation [11].

Tzanck smear cytology is particularly useful for the provisional diagnosis of PV, especially when biopsy is not feasible or in early stages of the disease [5]. In our study, cases of PV consistently demonstrated typical acantholytic cells. Conversely, other vesiculobullous lesions showed fewer keratinocytes, an absence of acantholytic cells, and a predominance of inflammatory cells, especially polymorphonuclear leukocytes. Based on the presence of acantholytic cells and clinical correlation, we suggested PV as a possible diagnosis and recommended immunofluorescence testing for confirmation. In contrast, bullous pemphigoid is typically characterized by abundant eosinophils in addition to inflammatory cells; acantholytic cells are generally absent [5]. The Tzanck smear provides rapid recognition of acantholysis, but histopathology remains the gold standard for confirming immunobullous disorders, with direct immunofluorescence and serological tests serving as adjuncts [12,13]. Interestingly, direct immunofluorescence has even been proposed for detecting immunoglobulin deposits directly on Tzanck smears [9].

Clinically, the features of venereal diseases may overlap with those of viral infections such as herpes simplex and varicella zoster virus (VZV). In our study, a clinically suspected case of varicella zoster exhibited numerous multinucleated giant cells along with scattered acantholytic cells on the Tzanck smear, supporting a cytological diagnosis of VZV infection. Similarly, a suspected bullous pemphigoid case revealed occasional clusters of multinucleated giant cells amid keratinocytes and inflammatory cells.

Tzanck smear cytology in PV shows predominantly acantholytic cells, which are large, round keratinocytes with basophilic cytoplasm, hyperchromatic nucleus, and perinuclear halo, often single cells, whereas, in VZV, acantholytic cells may be seen, but they are usually smaller and may show nuclear molding and chromatin margination. In PV, multinucleated giant cells are rare, and if present, they are due to cell fusion from acantholysis, while in VZV, multinucleated giant cells have a characteristic feature of multinucleation, nuclear molding, and margination of chromatin. These points give a clue to differentiate between PV and VZV cytomorphology.

The reliability of Tzanck smear depends not only on technique but also on the observer's expertise. Eryılmaz et al. assessed interobserver agreement and found substantial concordance, supporting the reproducibility of the test when performed systematically [3]. More recently, a blade-free modification of the Tzanck smear technique has been introduced to minimize patient discomfort while preserving diagnostic yield, reflecting how the test continues to evolve in modern dermatology [14].

Despite the widespread clinical use of Tzanck smear cytology, there remains a paucity of studies, particularly in the Indian context. Several Indian studies have systematically evaluated the diagnostic role of the Tzanck smear. Yaeen et al. studied 142 patients and demonstrated its value in vesiculobullous and erosive lesions [9]. Panwar et al. highlighted both its utility and pitfalls in 57 cases [8]. The most recent and largest Indian study by Chaudhari et al. included 224 cases and demonstrated sensitivity of 81% and specificity of 87.5% when compared with histopathology and desmoglein antibody status, while also describing detailed cytomorphological features of acantholytic cells [15]. A comparison of these studies with the present work is summarized in Table 4.

**Table 4:** Studies related to Tzanck smear from India

S. No.	Study	Number of cases	Key findings
1	Yaeen et al. (2015, India) [9]	142	Demonstrated diagnostic utility of Tzanck smear in vesicular, bullous, and erosive lesions; categorized into infections, immunologic disorders, genodermatoses, and spongiotic dermatitis.
2	Panwar et al. (2017, India) [8]	57	Reported diagnostic pitfalls and utility of Tzanck smear in cutaneous lesions, highlighting the need for clinicopathological correlation.
3	Chaudhari et al. (2025, India) [15]	224	Largest Indian series; reported sensitivity 81%, specificity 87.5%; detailed morphology of complete vs. incomplete acantholytic cells.
4	Present study (2025, India)	30	Evaluated the cytomorphological spectrum and diagnostic role of Tzanck smear in vesiculobullous lesions in Eastern Uttar Pradesh.

As an adjunct to routine histopathology, Tzanck smear cytology offers dermatologists a rapid, simple, and inexpensive diagnostic modality that can facilitate early clinical decision-making. A positive Tzanck smear allows for prompt initiation of treatment in infectious conditions, while a negative result can assist in excluding diseases such as pemphigus. Given its ease of use and minimal resource requirements, Tzanck smear cytology can serve as a valuable first-line investigation for vesiculobullous lesions.

Among the diagnostic investigations for vesiculobullous disorders, histopathology and direct immunofluorescence remain the most reliable methods, particularly for distinguishing between pemphigus and pemphigoid. Serological assays such as anti-desmoglein enzyme-linked immunosorbent assay (ELISA) provide additional sensitivity and are increasingly used as confirmatory tests. The use of immunofluorescence continues to play a crucial role in dermatology, particularly in differentiating between vesiculobullous disorders [16,17].

Our findings highlight the role of Tzanck smear cytology in minimizing the need for invasive procedures like biopsies, especially in busy tertiary care centers where quick diagnosis is crucial. This reinforces the relevance of this time-tested technique in contemporary dermatological practice.

The present study is limited by a small sample size from a single tertiary care center, which may affect the generalizability of the findings. Some patients were lost to follow-up, restricting histopathological or serological confirmation in all cases. Biopsy and serological tests, including anti-desmoglein antibody assays, were performed only in a subset of patients, limiting complete clinicopathological correlation. Despite these limitations, the study highlights the continuing relevance of Tzanck smear as a rapid, simple, and cost-effective diagnostic tool for vesiculobullous lesions, particularly in resource-limited settings.

## Conclusions

The Tzanck smear remains a valuable, rapid, and cost-effective diagnostic tool for evaluating vesiculobullous and certain infectious skin lesions. Its ability to identify characteristic cytological features, such as acantholytic cells in PV and multinucleated giant cells in viral infections, makes it especially useful for preliminary diagnosis when biopsy is not immediately feasible. When interpreted in conjunction with clinical findings, the Tzanck smear can guide the timely and appropriate management, thereby reducing the need for more invasive procedures. Despite being a longstanding technique, its role remains relevant and underutilized, particularly in resource-limited settings. This study highlights the importance of incorporating Tzanck smear cytology as a first-line diagnostic investigation in the workup of vesiculobullous dermatoses.
